# B Cell Profiling in Patients with Pemphigus Vulgaris

**DOI:** 10.32607/actanaturae.11890

**Published:** 2023

**Authors:** V. A. Abrikosova, Y. A. Mokrushina, L. A. Ovchinnikova, E. N. Larina, S. S. Terekhov, M. N. Baranova, Y. A. Lomakin, D. S. Balabashin, T. V. Bobik, E. N. Kaliberda, V. D. Knorre, M. V. Shpilevaya, T. K. Aliev, D. G. Deryabin, A. E. Karamova, A. A. Kubanov, M. P. Kirpichnikov, I. V. Smirnov

**Affiliations:** Shemyakin–Ovchinnikov Institute of Bioorganic Chemistry of the Russian Academy of Sciences, Moscow, 117997 Russian Federation; Faculty of Chemistry, Lomonosov Moscow State University, Moscow, 119991 Russian Federation; State Research Center of Dermatovenereology and Cosmetology, Moscow, 107076 Russian Federation; Faculty of Biology, Lomonosov Moscow State University, Moscow, 119991 Russian Federation; Endocrinology Research Center, Moscow, 117292 Russian Federation

**Keywords:** desmoglein 3, pemphigus vulgaris, targeted therapy, immunoligands

## Abstract

Pemphigus vulgaris is a severe, socially significant autoimmune disease associated with autoantibodies to the desmoglein 3 antigen. The disease affects all age groups, beginning at 18 years of age; the mortality rate of pemphigus can reach as high as 50%, depending on a patient’s age and a number of other factors. There is no highly selective or personalized therapy for pemphigus vulgaris at the moment. One of the well-known therapeutic approaches to the disease is to use rituximab, an anti-CD20 antibody that can help achieve B cell depletion in peripheral blood. To solve the problem of nonspecific elimination of B cells in patients with pemphigus vulgaris, it is reasonable to use specific immunoligands, their choice being based on an assessment of the level of autoantibodies specific to each of the fragments of desmoglein. In this work, the proportion of autoreactive B cells in patients diagnosed with pemphigus vulgaris is found to be 0.09–0.16%; a positive correlation was revealed between the antibody level and the number of autoreactive B cells to various fragments of desmoglein.

## INTRODUCTION

Pemphigus vulgaris is the most common form of bullous dermatosis, involving the appearance of blisters with serous content and a thin flaccid roof on unaltered skin and/or mucous membrane; once opened, they form painful erosions that do not heal for a long time.

According to state statistical monitoring data, 1.9– 2.4 new pemphigus vulgaris cases per 100,000 adult population (aged at least 18 years) are annually reported in Russia; the prevalence of the disease ranges from 4.8 to 6.3 cases per 100,000 population [1].

protein of desmosomes in stratified squamous epithelium, desmoglein 3 (Dsg3), play a key pathogenetic role in the development of pemphigus vulgaris [2]. The interaction between the autoantibodies and the extracellular domains of desmoglein 3 results in desmosomal degradation, followed by acantholysis (degenerative alterations in the stratum spinosum that manifests themselves as rupturing of intercellular bridges and lead to the formation of intraepidermal bullae) [3].

The standard therapy used for pemphigus vulgaris today consists of long-term administration of systemic corticosteroids, either as a monotherapy or in combination with other immunosuppressants, causing a number of serious adverse effects and being ineffective against forms of the disease that are resistant to systemic glucocorticoid (GC) therapy [4].

Monoclonal antibody-based drugs that enable personalized therapy of pemphigus vulgaris and other autoimmune diseases by targeting autoantibody-producing cells (B cells) are currently being developed to reduce the course dose of GCs. So far only rituximab is recommended for clinical application with pemphigus vulgaris patients, its active principle being chimeric monoclonal antibodies specific to B lymphocyte antigen CD20 [5]. However, the serious problem posed by rituximab use is that both the pathological (autoreactive) and normal B cells are systemically suppressed, thus leading to systemic immunodeficiency that is caused by a lack of circulating immunoglobulins. A series of cases have been reported when specific immunoactive agents were applied for a targeted elimination of pathological lymphocytes [6, 7]. Treatment specificity depends on the effectiveness of the interaction between the immunoactive drug and the target population of autoreactive B cells. It has been reported that there are differences in the level of antibodies specific to different desmoglein domains in patients diagnosed with pemphigus vulgaris [8, 9]. This fact can be used to enhance the immunotherapy specificity when performing targeted delivery of immunoactive agents comprising a specific variant of the desmoglein fragment. Immunosorption based on the elimination of autoreactive antibodies using highly selective immunosorbents from the blood of pemphigus vulgaris patients was proposed as a treatment option [10]. Therefore, it becomes necessary to determine the correlation between the antibody level and the proportion of autoreactive B cells targeting fragments of desmoglein and identify the specificity profile of the autoreactive B cells in pemphigus vulgaris patients.

## EXPERIMENTAL

The full-length recombinant extracellular fragment of human Dsg3 (EC1–EC5) and isolated domains EC1, EC2, and EC3–EC4 fused with the Fc fragment of human IgG1 were obtained in the CHO cell-based expression system using genetic constructs based on the pcDNA3.4 vector (Thermo Scientific, USA). Recombinant proteins were purified to remove the culture medium on a MabSelect SuRe column (GE Healthcare, USA). Protein purity was confirmed by size-exclusion chromatography and electrophoresis.

The total serum level of anti-Dsg3 antibodies in patients was characterized using the Anti-Desmoglein 3 ELISA IgG test kit (Euroimmun, Germany) and presented as relative activity units (RU/mL) according to the absorbance of the reference serum supplied together with the test kit. The resulting recombinant proteins were used to assess the immunoreactivity of serum samples from patients with pemphigus vulgaris by two-step competitive enzyme-linked immunosorbent assay (ELISA) [11]. Dsg3, as well as its fragments EC1, EC2, and EC3–EC4, fused with the Fc fragment of human IgG1 at a concentration of 1 μg/mL, as well as bovine serum albumin (BSA) at the same concentration for controlling nonspecific binding, were sorbed onto the wells of a polystyrene plate (Greiner Bio-One GmbH, Germany) overnight at +4°C. After removing the sorbed contents, the wells were washed once with phosphate-buffered saline (PBS) and blocked with a 0.1% casein solution. After the blocking, the wells were washed once with phosphate-buffered saline supplemented with 0.005% Tween-20 (PBST). The studied sera were diluted (1 : 100) in PBS supplemented with 1% BSA and incubated in a thermostated shaker at +24°C for 18 h at a rate of 200 rpm. Once the incubation was completed, the entire studied serum samples were transferred into the wells of the plate supplied with the reference test kit to repeatedly assess the level of antibodies that had not reacted with full-length Dsg3. Calibration samples with activities of 20 and 200 RU/mL were loaded to the additional wells and incubated using the same procedure as the one described previously (18 h, +24°C, 200 rpm). After the incubation, the plate was washed thrice with a PBST solution and antibodies specific to the kappa and lambda light chains of horseradish peroxidase-conjugated human antibodies were added. HRP-conjugated rabbit antibodies against full-length human IgG supplied with the reference test kit were added to the plate. After the 60-min incubation (+24°C, 200 rpm), the plate wells were washed thrice, supplemented with the substrate solution (tetramethylbenzidine, TMB), and incubated overnight in the dark for 30 min. The reaction was stopped by adding a 4 N phosphoric acid solution; the optical density (OD) in the wells was measured at a wavelength of 450 nm (OD_450_) on a plate reader. The OD values of the calibration curves with activities of 20 and 200 RU/mL recorded on the reference plate were used to plot a calibration curve that allowed one to determine the activity of each studied serum sample in relative activity units (RU/mL). The results were used to calculate the proportion (%) of autoreactive antibodies that specifically interacted with individual epitopes of the Dsg3 molecule and are detected by competitive ELISA:

[1 – (A_Pos_ – A_R_) / (A_Pos_ – A_Neg_)] × 100,

where A_R_ is serum activity after preincubation in the plate with an immobilized epitope protein, A_Pos_ is serum activity after preincubation with Dsg3 in the reference test kit, and A_Neg_ is serum activity after preincubation with BSA.

Venous blood samples collected from three patients with a clinical and laboratory diagnosis of L10.0 Pemphigus vulgaris were used in this study. All the patients had provided written informed consent to be included in the research; the study was conducted in compliance with current legal and ethical standards.

The number of autoreactive B cells was assessed by flow cytometry using biotinylated recombinant proteins (subdomains EC1, EC2, EC3, and EC4 fused with the constant domain of human immunoglobulin).

Peripheral blood mononuclear cells from pemphigus vulgaris patients and healthy donors were isolated using the Ficoll–Paque PLUS density gradient medium (GE Healthcare). The cells were washed, counted, resuspended in phosphate-buffered saline supplemented with Human Seroblock (Bio-Rad, USA), 0.5% BSA, and 2 mM EDTA (2 × 10^6^ cells per 100 μL of solution), and incubated on ice for 30 min. For a tetrameric complex to form, the preparations EC1-Fc, EC2-Fc, EC3-4-Fc, and Dsg3-Fc, purified and chemically biotinylated using the Sulfo-NHS-LC-Biotin reagent (Thermo Fisher Scientific), were mixed with Streptavidin- PE (Invitrogen, USA) and Streptavidin-Cy5 (Abcam, UK) at a 4 : 1 molar ratio and incubated at +4°C in the dark for 30 min. The tetrameric immune complex with Streptavidin-PE and tetrameric immune complex with Streptavidin-Cy5 were added to the cells being stained until concentrations of 4 nM and 10 nM, respectively, were achieved; the cells were incubated at +4°C under constant stirring for 15 min. Fluorescent anti-CD45-APC-Cy7 (1 : 300 dilution) (Sony, USA) and anti-CD19-PE-Cy7 (1 : 1000 dilution) (Biolegend, USA) antibodies and the SYTOXTMGreen fluorescent dead cell stain (1 : 1000 dilution) (Biolegend, USA) were then added to the samples being analyzed and additionally incubated at +4°C in the dark for 30 min. Next, the sam ples were washed with 0.5 mL of PBS supplemented with 2 mM EDTA. The fluorescence intensity was assessed on a ACEA Novocyte fluorimeter (ACEA Biosciences, USA).

## RESULTS AND DISCUSSION

**Fig. 1:**
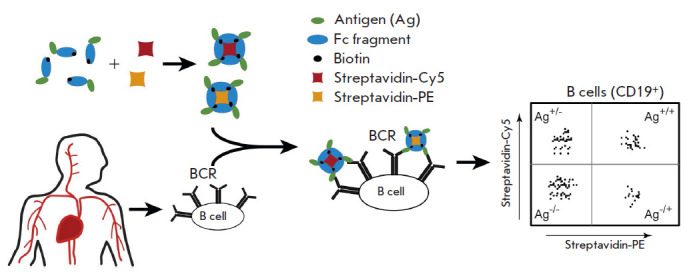
Schematic of B cell specificity assay workflow using double-positive antigen staining

The number of B cells autoreactive to desmoglein was determined in the venous blood of patients diagnosed with pemphigus vulgaris (P1–P3); blood from a healthy donor (HD) was used as a control sample. Mononuclear cells were isolated from all the samples to further perform staining and a flow cytometry assay (Fig. 1).

Serum samples for measuring the level of antibodies specific to Dsg3 and its fragments were collected separately (Table 1).

**Table 1:** The results of enzyme-linked immunosorbent assay of a pemphigus vulgaris patient’s serum using full-length desmoglein and its fragments as an antigen, represented in RU/ml

Antigen	P1	P2	P3
Dsg3	700	20	1500
EC1	588	-	300
EC2	203	-	450
EC3-4	98	-	750

One can see in Table 1 that the patients had different profiles of antibody response to the full-length protein and desmoglein 3 domains. Patient P2 was found to exhibit only a weak immune response to full-length Dsg3, equal to the diagnostically significant threshold value of 20 RU/mL, while patients P1 and P3 had a strong immune response that differed from the distal (EC1 and EC2) or proximal (EC3-4) extracellular domain of this protein.

**Fig. 2:**
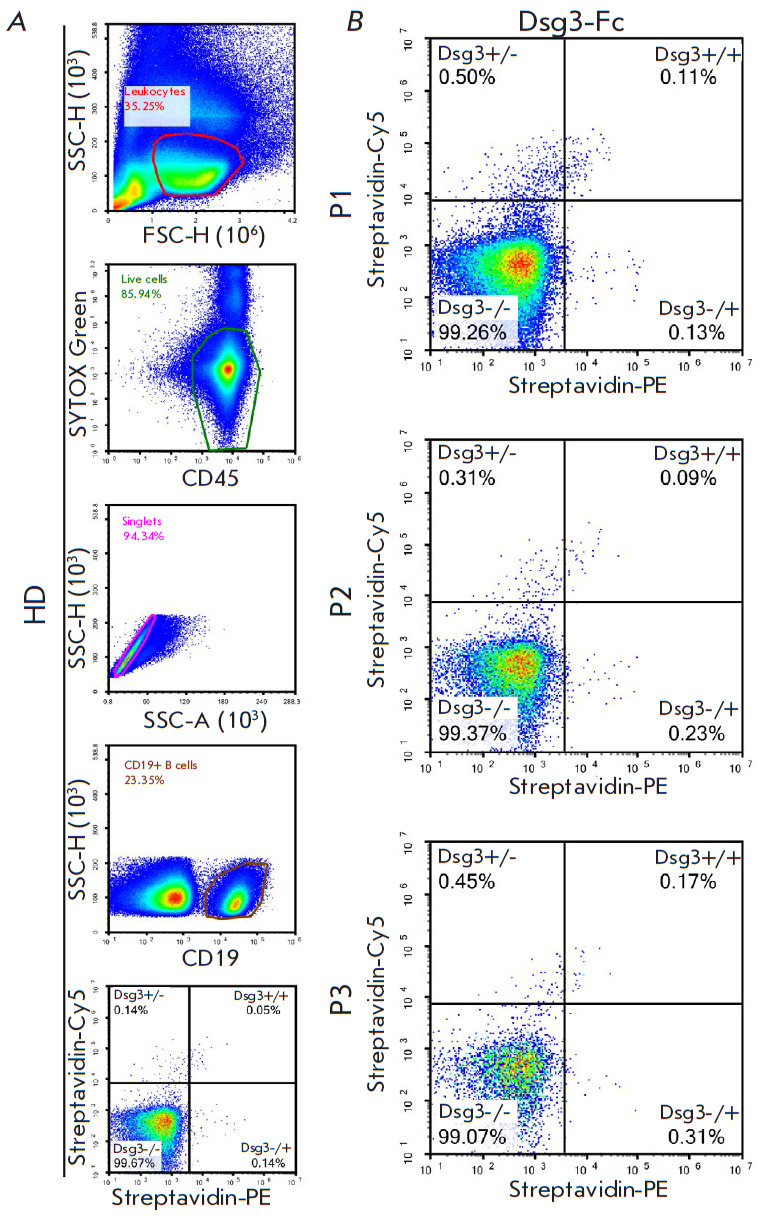
Flow cytometry analysis of a healthy donor (*A*) and pemphigus vulgaris patients (P1, P2, P3) to identify full-length Dsg3-specific B cells (*B*). The boundary condition for the plots in the Streptavidin-Cy5 and Streptavidin-PE scales were chosen as 0.05% positive events in the gate with "double positive" signal (+/+) for the control sample (healthy donor, HD)

In order to establish a correlation between the levels of antibodies specific to different desmoglein variants and the number of antigen-specific autoreactive B cells, the mononuclear cell fraction was stained with immunoactive ligands and antibodies specific to B cell surface antigens (CD19). It was shown earlier that the proportion of antigen-specific B cells is 0.05–0.5% of the entire B cell pool [12]. A tetrameric form of the immunoligand was designed using a fluorescently labeled streptavidin molecule (Fig. 1). A single molecule of the complex interacts with several molecules of the B cell receptor, thus increasing ligand avidity and, therefore, enhancing staining efficiency. Another key feature was employing the double-positive staining approach. Two streptavidin-fused antigenic complexes labeled with different fluorescent tags (phycoerythrin (PE) and cyanine dye Cy5) were used in this case. This approach significantly increased the level of specific staining of B cells. The staining mechanism reported in this study can be used to efficiently detect B cells targeting any identified antigens, including when searching for antibodies specifically bound to viral proteins, facilitating virus neutralization. The final scheme for staining/analyzing each sample involved the following: (i) isolating the area according to the cell size, (ii) isolating the area corresponding to live leukocytes after co-staining with the SYTOXTMGreen dye and anti-CD45-APC-Cy7 antibodies, (iii) isolating the area corresponding to single cells, (iv) isolating the area corresponding to CD19+ B cells, and (v) assessing double-positive antigen-specific B cells (Fig. 2).

**Fig. 3:**
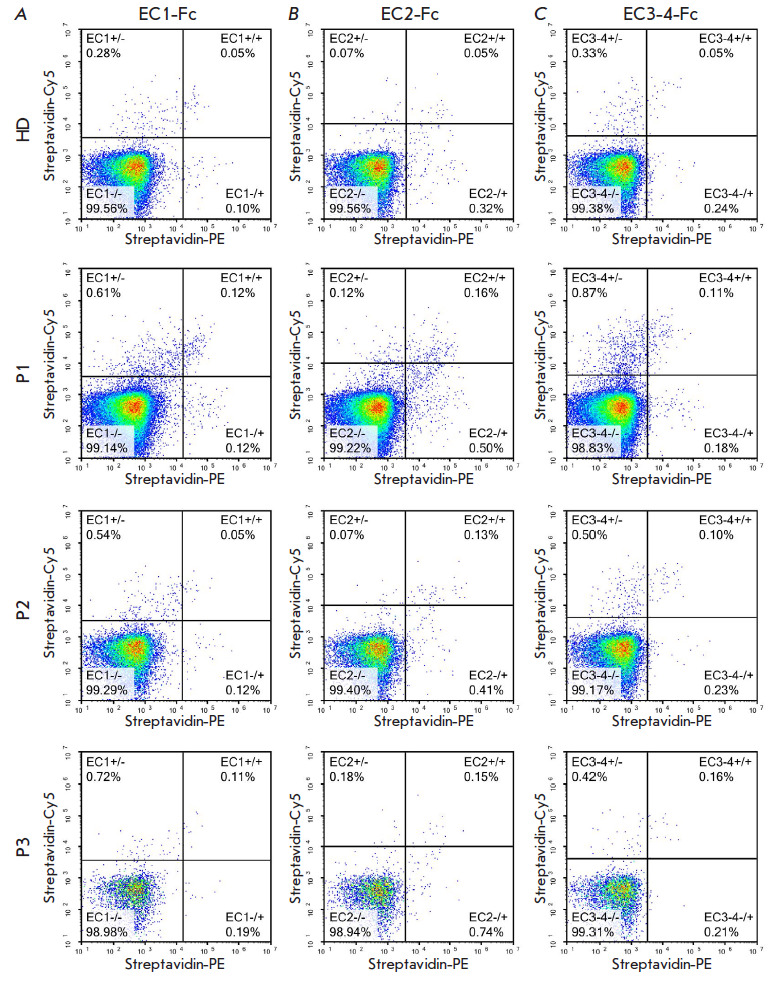
Flow cytometry analysis of a healthy donor (HD) and pemphigus vulgaris patients (P1, P2, P3) to identify EC1-specific (*A*), EC2-specific, and EC3-4-specific (*B*) B cells. The boundary condition for the plots in the Streptavidin-Cy5 and Streptavidin-PE scales were chosen as 0.05% positive events in the gate with "double positive" signal (+/+) for the control sample (healthy donor)

According to the results presented in Fig. 2B, the highest proportion of B cells specific to full-length desmoglein belonged to patient P3 while the lowest was true applied to patient P2; in general, these findings agree with the ELISA data (Table 1). The proportion of B cells specific to different domains of desmoglein was also different in all patients (Fig. 3).

The highest proportion of EC2-specific B cells was detected in patient P1; meanwhile, the proportion of cells EC1-specific and EC3-4-specific cells was lower: 0.12 and 0.11%, respectively. Patient P2 had no EC1- specific cells (the level being comparable to that of the control); the proportion of EC2-specific and EC3- 4-specific cells was 0.13 and 0.10%. In patient P3, the proportion of B cells specific to all the domains was comparable to that in patient P1; however, the proportion of EC2-specific and EC3-4-specific B cells was the highest (0.15 and 0.16%, respectively).

## CONCLUSIONS

**Fig. 4:**
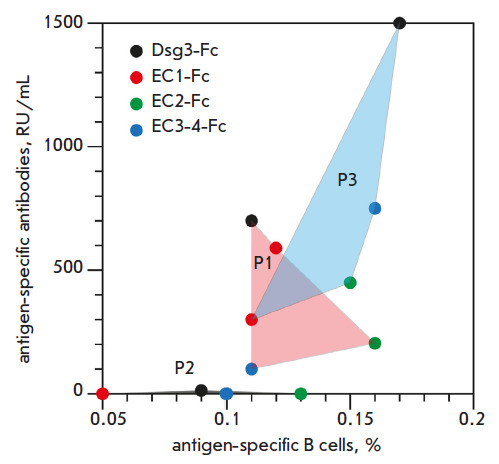
Profile analysis of the specificity of antibodies and B cells. Samples analyzed for full-length desmoglein 3 and its domains are color-coded. Lines connect values related to the same patient

Hence, the profile analysis of the specificity of antibodies and B cells showed that there exists a generally positive correlation between the blood titer of specific antibodies and the proportion of antigen-specific B cells (Fig. 4).

Meanwhile, the proportion of autoreactive B cells in pemphigus patients was in the range of 0.09–0.16%. A noticeable level of nonspecific binding was also detected in a healthy donor (0.05%); however, the double-positive antigen staining approach has made it possible to determine the antigen-specificity profile of B cells in pemphigus vulgaris patients. The results are important in elaborating a strategy of personalized pemphigus therapy using cytotoxic immunoligands based on recombinant desmoglein domains fused with the Fc fragment of human IgG1. It is likely that patient P2 will be insensitive to therapy with EC1-Fc, while one may expect the elimination of autoreactive B cells when using EC2-Fc and EC3-4-Fc in patients P1 and P3, respectively. B cell profiling in patients with autoimmune diseases, and pemphigus vulgaris in particular, opens up broad prospects for choosing a personalized treatment strategy.
